# How has the COVID-19 Pandemic Affected Diabetes Self-Management in People With Diabetes? - A One-Year Follow-Up Study

**DOI:** 10.3389/fcdhc.2022.867025

**Published:** 2022-08-17

**Authors:** Kasper Olesen, Lene Eide Joensen, Kristoffer Panduro Madsen, Ingrid Willaing

**Affiliations:** ^1^ Health Promotion Research, Copenhagen University Hospital – Steno Diabetes Center Copenhagen, Herlev, Denmark; ^2^ Section of Health Services Research, Department of Public Health, Copenhagen University, Copenhagen, Denmark

**Keywords:** diabetes self-management, diabetes distress, psychological distress, lifestyle, corona virus (COVID-19), SARS-CoV-2

## Abstract

**Background and Aim:**

In Denmark, the COVID-19 pandemic resulted in two lockdowns, one from March to May 2020 and another from December 2020 to April 2021, which had severe impact on everyday life. The aim of this study was to explore changes in diabetes self-management behaviors during the pandemic and to examine how specific population characteristics were associated with changes in diabetes management.

**Methods and Participants:**

In a cohort study from March 2020 to April 2021, two online questionnaires were collected from a total of 760 people with diabetes. Descriptive statistics were used to assess the proportion of participants experiencing improvements, deterioration, and status quo in diabetes self-management during the pandemic. Using logistic regressions, baseline characteristics were explored as potential predictors of change.

**Results:**

Approximately half of the participants reported that they experienced lower physical activity in April 2021 compared to before the pandemic, approximately one fifth reported diabetes self-management to be more difficult than prior to the pandemic, and one fifth reported eating more unhealthily than before the pandemic. Some participants reported higher frequency of high blood glucose levels (28%), low blood glucose levels (13%) and more frequent blood glucose variability (33%) compared to before. Easier diabetes self-management was reported by relatively few participants, however, 15% reported eating more healthily, and 20% reported being more physically active. We were largely unable to identify predictors of change in exercise activities. The few baseline characteristics identified as predictors of difficulties in diabetes self-management and adverse blood glucose levels due to the pandemic were sub-optimal psychological health, including high diabetes distress levels.

**Conclusion:**

Findings indicate that many people with diabetes changed diabetes self-management behaviors during the pandemic, mostly in a negative direction. Particularly high diabetes distress levels in the beginning of the pandemic was a predictor of both positive and negative change in diabetes self-management, indicating that people with high diabetes distress levels could potentially benefit from increased support in diabetes care during a period of crisis.

## Introduction

In early 2020, the COVID-19 disease spread worldwide and was declared a global pandemic by the World Health Organization (WHO) on March 11 (1). The consequences of COVID-19 infection were largely unknown at the time, but it was quickly established that the disease could cause severe respiratory infections and potentially be fatal (2). Mass-media coverage reported high mortality levels in some regions of developed countries, and societies across the globe quickly implemented measures to prevent spread of the disease. Among other challenges, it was suggested that the pandemic and its accompanying societal changes could result in significant consequences for access to high quality health care. Moreover, it was reported that people with diabetes were at increased risk of severe disease if infected with COVID-19 (3).

In Denmark, all schools and childcare services were closed on March 12, 2020, employees in the public sector with noncritical functions were ordered to work from home and gatherings of more than ten people were prohibited, resulting in many people not going to work on a daily basis and temporary closure of non-critical public facilities such as cinemas, restaurants, and sport facilities (4). During the following months, restrictions were gradually lifted; however, some restrictions were reintroduced the following winter due to an increased incidence of COVID-19 in the population (4). From December 2020 to April 2021, a second lockdown was enforced due to renewed widespread disease in the population.

For people with diabetes, complex diabetes self-management is necessary to avoid adverse acute and long-term consequences of the illness and is, for many people, an integrated part of daily life (5, 6). A primary goal in the management of diabetes is to always obtain near-normal blood sugar levels for which exercise and a healthy diet are among the integral components (5, 6). A variety of factors influencing diabetes self-management has been identified including age, gender, educational attainment, diabetes type and duration, and psychological health. However, consistency is lacking across studies and most of the variance in diabetes self-management remains unexplained (7). This indicates that factors influencing diabetes self-management are not fully understood and may vary across time and setting.

Research on the impact of the COVID-19 pandemic and diabetes management has suggested that many people experienced difficulties in managing their diabetes due to the pandemic. Thus, deterioration in healthy lifestyle, i.e., less exercise and unhealthier diet have been observed in various populations (8–13), and according to one study particularly in women (9). A single study found improvements in diabetes self-management in a population characterized by young participants with high educational level (14), whereas a recent qualitative study found improvements in diabetes self-management (15). A recent meta-analysis analyzed the body of literature on COVID-19 and blood glucose levels in people with type 1 and type 2 diabetes and concluded that the studies were heterogenous in their findings with many studies being inconclusive (16). Some studies, however, reported higher blood glucose levels either in combination with weight gain (17, 18) or independent of weight gain (19). While previous studies have suggested that changes in diabetes self-management due to the pandemic have been negative overall, it is largely unknown what characterizes people with diabetes who have experienced either improved or deteriorated diabetes self-management behaviors during the pandemic. Research has established that people with diabetes distress experience difficulties in managing diabetes (20–22). The potential psychosocial burden of the pandemic and disruption in everyday life activities by societal restriction may have constituted a new challenge for people with diabetes in maintaining their diabetes management (20–22). Whilst the restrictions for many may have been perceived as a burden, they could simultaneously offer opportunities for improved diabetes self-management (15). Knowledge about characteristics that facilitated or reduced optimal diabetes care during the pandemic may give the opportunity to move away from “one-size-fits-all solutions” and in times of crisis to focus resources at those with the highest risk of being negatively affected by such disruption.

For these reasons, the aim of this study was to explore changes in self-reported ability to effectively manage diabetes, including changes in diet, exercise, and blood glucose levels from the beginning and one year into the pandemic. Another aim was to examine how selected population characteristics were associated with positive as well as negative changes in diabetes self-management during the pandemic.

## Materials and Methods

### Study Design

In March 2020, a cohort was established by inviting 2,430 adults (aged >18 years) with diabetes to participate in an online questionnaire-based survey, hereafter referred to as the ‘baseline questionnaire’. In a follow-up questionnaire one year after the initiation of the survey, responders from the initial survey were invited to complete a new questionnaire, referred to hereafter as the ‘follow-up questionnaire’, providing information about their experienced changes in diabetes management behaviors during the pandemic and their everyday life during the last year of the COVID-19 pandemic. Participants were recruited from two user panels situated at, respectively, Steno Diabetes Center Copenhagen and the Danish Diabetes Association. Together, the panels represent people with type 1 or type 2 diabetes from across Denmark.

Questionnaires were sent to potential participants’ e-mail addresses, and informed consent was obtained digitally from all participants. Upon sign-up to each of the user panels, participants had agreed to be contacted for research purposes. For this analysis, we exclusively utilized data from participants responding to both the baseline and the follow-up questionnaire. Among these participants, characteristics from the baseline questionnaire was compared to behavior changes reported in the follow-up questionnaire, approximately one year after the baseline survey. Moreover, descriptive data from both baseline and follow-up were presented as well. Recruitment strategy and baseline characteristics have been reported in detail elsewhere (23).

### Included Measures

Information about age, gender, education, employment status, cohabitation status, type of diabetes, diabetes duration, diabetes complications, and health status was obtained from the baseline questionnaire (23). Likewise, psychosocial data on diabetes distress (2-item Diabetes Distress Scale (DDS-2)) (24), quality of life (25) and worries related to diabetes and the pandemic (23) were collected from the baseline questionnaire. Diabetes distress and quality of life were measured on continuous scales; worries about diabetes were measured by a set of dichotomous variables. Baseline data and measures have been described in detail elsewhere (23). Variables measuring perceived impact of the COVID-19 pandemic on diabetes self-management in the follow-up questionnaire were developed by Fisher and colleagues (13) and translated into Danish for this study. Wordings and meanings of the adopted questions were tested among people similar to the participants. After minor adjustments, the interviewees did not indicate further problems related to comprehensibility and unambiguity of the questions. Impact of the pandemic on difficulties in diabetes management was assessed by the question, “Compared to before the coronavirus pandemic, how has your diabetes management changed?” It was assessed by a 7-point scale from “significantly easier”, “moderately easier”, “slightly easier”, “no change”, “slightly harder”, “moderately harder”, and “significantly harder,” with the opportunity to indicate “no change” using the middle option (13). A similar item asked about changes in the amount of exercise, using a 7-point scale from “much less” to “much more” (13). Furthermore, a new item was developed measuring changes in diet ranging from “much healthier” to “much unhealthier”. Perceived impact of the coronavirus pandemic on blood glucose levels was assessed using 7-point scales asking the respondent to indicate if they experienced major changes regarding frequency of: high blood glucose, low blood glucose and larger glucose variability with response options ranging from “a lot more frequent” to “a lot less frequent” (13). Medication taking was measured with a similar item but with only five response options: “a lot more regularly”, “a little more regularly”, “no change”, “a little less regularly”, and a lot less regularly. Exact wording of the outcome variables and their response options is available in **Supplementary Material**.

### Statistical Analysis

Population characteristics and perceived changes in diabetes management behaviors were explored using descriptive statistics. The data were inappropriate for cumulative logit models due to violation of the proportional odds assumption. Instead, baseline predictors of change in diabetes management were analyzed using nominal logistic regressions with independent variables measured at baseline and categorized outcome variables measured at follow up. The 7-point scales used as outcomes were reversed if needed to let low values consistently represent desirable status and collapsed into three levels 1–3 (positive change), 4 (unchanged), 5–7 (negative change). The “unchanged” category was used as reference.

All estimates were adjusted for age, gender, education, employment status, diabetes type, diabetes duration, diabetes-specific complications, and co-morbidities. The models were not adjusted for psychosocial variables, i.e., DDS-2, quality of life and worries due to similarities between the concepts and risk of over-adjustment. Thus, impact of psychosocial factors was estimated in independent models, each with a single psychosocial factor added. All analyses were carried out using SAS Enterprise Guide 7.1. The level of significance was p < 0.05.

## Results

Out of 1,396 individuals who replied to the baseline questionnaire, 760 (54%) also responded to the follow-up questionnaire.

**Table 1** shows characteristics of the study population at baseline. Most participants were older than 65 years, men (58%), and had completed a higher education (<5 years) (58%). Almost two thirds had type 2 diabetes, had long diabetes duration (50% > 15 years) and almost one out of five had one or more complications to diabetes. More than half (55%) had at least one additional chronic disease, whereas one out of ten reported at least one mental disorder. Most participants (71%) reported high quality of life, whereas (78%) scored less than two on the DDS-2 indicating little or no diabetes distress. Almost two thirds were worried about being overly affected due to their diabetes if infected with COVID-19, and one third was worried about not being able to manage their diabetes properly if infected by COVID-19.

**Table 1:** Baseline characteristics of the study population (N = 760).

Characteristic	Frequency (%)
**Gender**	
Male	439 (57.8)
Female	321 (42.2)
**Age (years)**	
Years (P25, Median, P75)	(57, 66, 72)
**Educational attainment**	
Primary	44 (5.8)
Secondary	243 (32.0)
Higher education (<5 years)	441 (58.0)
Higher education (>= 5 years)	32 (4.2)
**Employment**	
Employed	251 (33.0)
Retired	453 (59.6)
Other	56 (7.4)
**Diabetes type**	
Type 1	262 (34.5)
Type 2	498 (65.5)
**Diabetes duration**	
Years (P25, Median, P75)	(8, 15, 26)
**Complications to diabetes**	
0 complications	611 (80.4)
1+ complications	149 (19.6)
**Chronic diseases (other than diabetes)**	
0 chronic diseases	341 (44.9)
1+ chronic diseases	419 (55.1)
**Mental disorder**	
None	673 (88.6)
1+ mental disorder	87 (11.4)
Quality of Life*	
Suboptimal quality of life	224 (29.5)
High quality of life	536 (70.5)
Diabetes distress*	
Little to no DDS (score <2)	589 (77.5)
Moderate to high DDS (score >=2)	171 (22.5)
**Overly affected due to diabetes if infected**	
Not worried	293 (38.6)
Worried	467 (61.5)
**Unable to manage diabetes if infected**	
Not worried	521 (68.6)
Worried	239 (31.4)

*Continuous variable categorized exclusively for this table for the purpose of meaningful presentation.

**Table 2** shows the distributions of perceived changes in difficulties in diabetes self-management, medication taking, diet, physical activity, high blood glucose levels, low blood glucose levels and blood glucose variability measured one year after baseline. Approximately three quarters of the participants reported unchanged difficulties in managing diabetes, almost none (3%) reported that diabetes management was easier during the pandemic whereas the remaining participants (21%) reported diabetes management to be harder. Almost all participants (93%) reported unchanged medication taking. Most of the participants experienced unchanged diet (62%) and blood glucose levels (65-, 62-, 79%) whereas a minority (29%) experienced unchanged physical activity. Among people who experienced changes, difficulties in managing diabetes were more frequent in all categories – particularly regarding high and low blood glucose levels and blood glucose variability, where less than 8% experienced improvements in one or more of the measures. In all, 13- to 33% experienced more frequent low and high blood glucose levels and blood glucose variability.

**Table 2:** Self-reported changes in diabetes self-management behaviors.

Events	Proportion (%) responses in categories						
Diabetes management	Much Easier	Somewhat Easier	Easier	Unchanged	Slightly Harder	Somewhat Harder	Much Harder
Type 1 diabetes	0.4	1.2	3.1	71.8	20.2	3.1	0.4
Type 2 diabetes	0.8	0.4	1.0	76.3	15.3	4.6	1.6
All	0.7	0.7	1.7	74.7	17.0	3.1	1.2
**Medication taking**		Much more regularly	Slightly more regularly	Unchanged	Slightly less regularly	Much less regularly	
Type 1 diabetes			3.1	92.8	3.1	1.2	
Type 2 diabetes		0.2	0.6	92.8	2.1	4.2	
All		0.1	1.5	92.8	2.5	3.2	
**Diet**	Much Healthier	Somewhat Healthier	Healthier	Unchanged	Slightly Unhealthier	Somewhat Unhealthier	Much Unhealthier
Type 1 diabetes	0.0	2.7	7.3	67.2	18.3	4.2	0.4
Type 2 diabetes	2.2	3.4	11.9	59.8	17.3	5.2	0.2
All	1.5	3.2	10.3	62.4	17.6	4.9	0.3
**Physical activity**	Much More	Somewhat More	Slightly More	Unchanged	Slightly Less	Somewhat Less	Much Less
Type 1 diabetes	1.2	4.6	13.0	30.5	26.3	16.4	8.0
Type 2 diabetes	1.2	7.6	12.5	28.3	20.3	21.9	8.2
All	1.2	6.6	12.6	29.1	22.4	20.0	8.2
**Blood sugar levels**	Much Less	Somewhat Less	Slightly Less	Unchanged	Slightly More	Somewhat More	Much More
**High blood sugars**							
Type 1 diabetes	0.8	2.3	3.8	60.3	23.7	9.2	0.0
Type 2 diabetes	1.4	1.4	4.2	67.3	20.1	4.8	0.8
All	1.2	1.7	4.1	64.9	21.3	6.3	0.5
**Blood sugar variability**							
Type 1 diabetes	0.4	0.8	3.8	54.6	27.9	10.7	1.9
Type 2 diabetes	1.0	1.2	2.0	66.5	22.9	5.8	0.6
All	0.8	1.1	2.6	62.4	24.6	7.5	1.1
**Low blood sugars**							
Type 1 diabetes	1.2	1.9	4.6	66.8	22.1	3.1	0.4
Type 2 diabetes	3.4	1.0	3.6	84.7	6.2	1.0	0.0
All	2.6	1.3	4.0	78.6	11.7	1.7	0.1

Now compared to before the pandemic (N = 760).

Bold values indicate statistically significant predictors.

**Tables 3A**, **3B** show regression coefficients predicting whether the pandemic had made it, respectively, easier (3A) or harder (3B) to manage diabetes according to participant reporting one year after baseline.

**Table 3A:** Predictors of reporting improved diabetes self-management behaviors during the pandemic.

	Less frequent high blood sugar	Less frequent blood sugar variability	Less frequent low blood sugar	Healthier diet	More physical activity
Characteristics (measure)	**OR (95% CI)**	**OR (95% CI)**	**OR (95% CI)**	**OR (95% CI)**	**OR (95% CI)**
Female (ref male)	1.19 (0.65; 2.18)	1.18 (0.56; 2.49)	1.35 (0.77; 2.38)	1.39 (0.89; 2.18)	1.34 (0.86; 2.08)
Age (years)	0.99 (0.96; 1.03)	1.00 (0.96; 1.05)	0.98 (0.95; 1.02)	1.01 (0.98; 1.04)	0.98 (0.95; 1.01)
Secondary education (ref primary)	0.71 (0.24; 2.09)	1.20 (0.25; 5.76)	0.57 (0.21; 1.55)	1.84 (0.60; 5.68)	1.02 (0.37; 2.80)
Higher education <5 years (ref: primary)	0.51 (0.18; 1.46)	0.77 (0.17; 3.57)	0.52 (0.20; 1.35)	2.07 (0.69; 6.21)	1.41 (0.53; 3.77)
Higher education >= 5 years (ref primary)	1.45 (0.36; 5.92)	3.31 (0.53; 20.71)	0.20 (0.02; 1.84)	3.13 (0.75; 13.10)	1.31 (0.31; 5.50)
Retired (ref working)	0.96 (0.42; 2.20)	1.25 (0.43; 3.65)	1.26 (0.57; 2.77)	0.66 (0.35; 1.23)	0.91 (0.49; 1.68)
Other (ref working)	1.13 (0.38; 3.31)	1.50 (0.38; 5.97)	1.77 (0.68; 4.64)	0.90 (0.37; 2.19)	0.65 (0.26; 1.61)
Type 2 (ref type 1)	0.98 (0.45; 2.16)	0.57 (0.23; 1.42)	1.03 (0.48; 2.18)	**1.92 (1.05; 3.51)**	1.43 (0.81; 2.52)
Diabetes duration (years)	0.99 (0.97; 1.02)	0.99 (0.96; 1.02)	1.01 (0.99; 1.03)	0.99 (0.97; 1.01)	0.99 (0.98; 1.01)
complications (ref no complications)	1.84 (0.91; 3.71)	0.77 (0.28; 2.16)	1.05 (0.52; 2.14)	1.54 (0.90; 2.64)	1.18 (0.67; 2.07)
1+ chronic illness (ref: none)	0.95 (0.52; 1.74)	1.25 (0.60; 2.62)	1.51 (0.85; 2.68)	1.09 (0.70; 1.70)	0.97 (0.63; 1.50)
1+ mental disorder (ref: none)	1.18 (0.50; 2.75)	0.41 (0.09; 1.85)	0.79 (0.33; 1.88)	**2.57 (1.40; 4.71)**	0.72 (0.37; 1.41)
Quality of life (1-10)	0.94 (0.81; 1.10)	0.97 (0.79; 1.18)	**0.85 (0.74; 0.97)**	0.95 (0.84; 1.06)	1.02 (0.90; 1.16)
DDS (1-6)	**1.58 (1.17; 2.13)**	**1.67 (1.15; 2.42)**	**1.46 (1.12; 1.92)**	**1.80 (1.42; 2.27)**	1.07 (0.84; 1.38)
Worried. overly affected (y/n)	0.86 (0.47; 1.55)	0.78 (0.38; 1.62)	0.84 (0.48; 1.46)	1.45 (0.92; 2.29)	1.06 (0.68; 1.64)
Worried. unable to manage diabetes (y/n)	1.35 (0.72; 2.53)	1.78 (0.83; 3.82)	**2.25 (1.27; 4.00)**	1.07 (0.66; 1.74)	1.04 (0.65; 1.66)

Adjusted for gender, age, educational attainment, occupational status, diabetes type, diabetes duration, diabetes complications, and comorbidities. Improved compared to unchanged behavior. (N = 760).

Bold values indicate statistically significant predictors.

**Table 3B:** Predictors of reporting worse diabetes management behaviors during the pandemic.

	More frequent high blood sugar	More frequent blood sugar variability	More frequent low blood sugar	Unhealthier diet	Less physical activity	Harder to manage diabetes
Characteristics (measure)	**OR (95% CI)**	**OR (95% CI)**	**OR (95% CI)**	**OR (95% CI)**	**OR (95% CI)**	**OR (95% CI)**
Female (ref male)	1.27 (0.90; 1.79)	1.21 (0.87; 1.68)	**1.62 (1.02; 2.58)**	**1.83 (1.25; 2.66)**	1.30 (0.91; 1.87)	1.39 (0.96; 2.01)
Age (years)	1.00 (0.98; 1.02)	0.98 (0.96; 1.00)	1.02 (0.99; 1.04)	0.97 (0.95; 0.99)	0.99 (0.97; 1.01)	0.97 (0.95; 0.99)
Secondary education (ref primary)	1.61 (0.72; 3.58)	1.51 (0.74; 3.09)	1.86 (0.53; 6.58)	0.92 (0.43; 1.98)	0.75 (0.35; 1.60)	1.07 (0.47; 2.40)
Higher education <5 years (ref: primary)	1.23 (0.56; 2.68)	0.83 (0.41; 1.69)	1.16 (0.33; 4.04)	0.77 (0.37; 1.62)	0.85 (0.40; 1.77)	1.00 (0.45; 2.20)
Higher education >= 5 years (ref primary)	1.17 (0.38; 3.61)	1.26 (0.45; 3.51)	1.07 (0.22; 5.19)	1.14 (0.38; 3.44)	1.02 (0.34; 3.06)	1.81 (0.61; 5.37)
retired (ref working)	0.90 (0.56; 1.44)	1.00 (0.63; 1.58)	0.80 (0.42; 1.52)	0.79 (0.47; 1.32)	0.79 (0.48; 1.31)	0.67 (0.40; 1.10)
other (ref working)	0.97 (0.50; 1.87)	1.09 (0.58; 2.03)	0.89 (0.39; 2.06)	0.96 (0.48; 1.90)	0.89 (0.45; 1.77)	0.66 (0.33; 1.33)
Type 2 (ref type 1)	0.73 (0.47; 1.12)	0.68 (0.45; 1.04)	0.20 (0.11; 0.34)	**1.73 (1.05; 2.85)**	1.18 (0.75; 1.84)	1.59 (0.97; 2.59)
Diabetes duration (years)	1.00 (0.99; 1.01)	1.00 (0.99; 1.01)	1.00 (0.98; 1.01)	1.00 (0.98; 1.01)	0.99 (0.98; 1.01)	1.01 (1.00; 1.03)
complications (ref: no comp.)	1.23 (0.80; 1.89)	**1.55 (1.03; 2.33)**	1.31 (0.76; 2.28)	1.49 (0.92; 2.40)	1.28 (0.82; 2.00)	1.42 (0.89; 2.25)
1+ chronic illness (ref: none)	0.99 (0.71; 1.39)	1.08 (0.78; 1.50)	1.04 (0.66; 1.65)	1.45 (0.99; 2.13)	1.24 (0.88; 1.77)	1.22 (0.84; 1.78)
1+ mental disorder (ref: none)	0.92 (0.54; 1.57)	0.84 (0.51; 1.39)	0.92 (0.46; 1.88)	1.23 (0.69; 2.17)	0.63 (0.37; 1.08)	**1.97 (1.18; 3.29)**
Quality of life (1-10)	**0.88 (0.80; 0.96)**	**0.85 (0.78; 0.93)**	0.93 (0.83; 1.05)	**0.89 (0.81; 0.98)**	**0.86 (0.78; 0.95)**	**0.84 (0.76; 0.92)**
DDS (1-6)	**1.51 (1.25; 1.82)**	**1.60 (1.33; 1.92)**	**1.47 (1.16; 1.85)**	**1.56 (1.27; 1.91)**	1.11 (0.91; 1.35)	**1.96 (1.61; 2.39)**
Worried. overly affected (y/n)	**1.60 (1.12; 2.28)**	**1.56 (1.11; 2.18)**	1.25 (0.77; 2.03)	1.45 (0.98; 2.13)	1.11 (0.78; 1.57)	**2.32 (1.54; 3.49)**
Worried. manage diabetes (y/n)	1.03 (0.72; 1.49)	1.30 (0.92; 1.85)	**1.87 (1.18; 2.98)**	1.17 (0.78; 1.75)	0.87 (0.59; 1.27)	**1.93 (1.31; 2.85)**

Adjusted for gender, age, educational attainment, occupational status, diabetes type, diabetes duration, diabetes complications, and comorbidities. Worsened compared to unchanged behavior. (N = 760). Bold values indicate statistically significant predictors

Shown in **Table 3A**, participants with less frequent high blood glucose levels, less frequent blood glucose variability and less frequent low blood glucose levels were characterized by higher levels of diabetes distress compared to participants experiencing no change in blood glucose levels. Participants who reported healthier eating were more likely to have a mental disorder and to have type 2 diabetes. Additionally, participants who experienced less frequent low blood glucose levels had lower quality of life and were more often worried about their diabetes management during the pandemic compared to participants experiencing no change in blood glucose levels. Due to the low number of participants reporting easier diabetes management (n=34), more regularly medication taking (n = 12) and less regularly medication taking (n =45) medication taking, the study did not offer the opportunity to explore predictors of these kind of changes.

As shown in **Table 3B**, experiences of more frequent high blood glucose levels and more frequent blood glucose variability were associated with lower quality of life, higher diabetes distress and worries about being overly affected by the COVID-19 in case of infection. Experiences of more frequent low blood glucose levels were associated with higher diabetes distress and being worried about management of diabetes during the pandemic. Participants who reported eating more unhealthily were more likely to be female, have type 2 diabetes and have lower quality of life.

## Discussion

The onset of the COVID-19 pandemic from March 2020 had significant impact on everyday life among people with diabetes as well as on their diabetes self-management the following year. In this study of 760 individuals with type 1 or type 2 diabetes, several changes in diabetes self-management were observed. However, most participants reported unchanged diabetes self-management across all investigated aspects, except for physical activity for which many reported less activity. Negative impact of COVID-19 was dominating compared to positive impact in almost all investigated aspects of diabetes self-management, including impact on difficulties in diabetes management, eating unhealthily and doing less physical activity. Negative changes were also reported in terms of more frequent sub-optimal blood glucose levels. Almost one out of four participants reported diabetes management to be harder during the pandemic; very few reported it to be easier. This means that despite home-based working and cancelled social activities with better opportunities for planning and performing diabetes self-management, only very few participants considered the situation advantageous for diabetes management. In line with this, most studies find that diabetes management has been increasingly difficult and less effective due to the pandemic (8–11, 26, 27) whereas a single study on young, highly educated people demonstrated improved diabetes management (14). Likewise most studies report negative impact on specific self-management behaviors, i.e., less exercise and more unhealthy diet during the pandemic across various populations (12, 18, 28–32). Regarding changes in blood glucose levels during the pandemic, previous findings are mixed and improvements have been found particularly in studies of people with type 1 diabetes (15, 33). Our finding that less physical activity was the most frequently reported behavior change is not surprising, as the physical activity level was likely to be affected when organized sports activities were shut down during the pandemic. Furthermore, for people who were working from home, transport to work and physical activity as a part of the workday was not “automatically” happening. The results suggest that the lack of exercise due to COVID-19 restrictions was not compensated for by the participants by introducing new forms of appropriate exercise. This finding is in line with a systematic review of exercise among older adults who, during the lockdown, performed less exercise or presented with more sedentary behavior. Most of the identified studies of behavior change during the lockdown presented the same findings (12, 34). Likewise, maintaining an unhealthier diet was also reported in some studies whereas none reported improved diet because of the lockdown (33). Lower level of physical activity in the study population combined with overall difficulties in managing diabetes is likely to have influenced blood glucose levels (34) which may explain the increased frequency of high and low blood glucose levels as well as variability in blood glucose levels during the pandemic.

Overall, our analyses showed that sub-optimal psychosocial health was associated with negative impact on diabetes self-management behaviors and frequency of adverse blood glucose levels. Particularly diabetes distress at baseline was associated with changes in diabetes self-management behaviors during the pandemic. Importantly, higher diabetes distress was simultaneously associated with lowered and increased frequency of adverse blood glucose levels. Research has shown associations between psychosocial health and diabetes self-management. For example, research before the pandemic had already established strong evidence for associations between diabetes distress and diabetes self-management (20, 35). Improved diabetes self-management among some participants with high diabetes distress may be a consequence of successful support from health care professionals and/or relatives combined with room for improvements in diabetes self-management behaviors in this sub-population of people with diabetes distress. This suggests that people with diabetes distress constitute a heterogenous group with different needs for support. We were largely unable to identify characteristics of people with changed level of physical activity indicating that people who changed level of physical activity was a heterogenous group with no common characteristics. People in jobs, among whom many were sent home, were not significantly more prone to changes in diabetes self-management during the lockdown.

A key strength of the present study is the individual level linkage between survey responses in the early stages of the pandemic and follow up responses one year later. This provided the opportunity to include factors measured at baseline in the first week of the first lockdown – free of influence from future events - to the changes reported at follow up. Another strength of the study is the high number of participants with type 1 and type 2 diabetes included in the study population. The inclusion of validated measures in the questionnaire ensured high internal validity of the self-reported predictors.

Limitations include that due to relatively few participants reporting improvements in diabetes self-management behaviors, the study lacked statistical power to sufficiently explore predictors of improved diabetes management during the pandemic. Mechanisms towards improved diabetes management during pandemics as well as other societal crises should be explored with different study designs. As with any self-report method, recall bias may have influenced the data and findings of the study, particularly when asking to events more than a year back in time in the follow-up questionnaire and accuracy of the responses may be reduced. Therefore, we did not ask participants to give exact measures of changes in their activities, but merely requested participants to report trends, e.g., much less, somewhat less, slightly less, unchanged. The exact proportion of people reporting change should be interpreted with caution. Diabetes self-management behaviors are, however, an integral part of everyday life for people with diabetes, and it is likely that significant behavior changes will be remembered. The beginning of the pandemic and national lockdown was a watershed moment for many Danes, which makes the everyday life and events of the time easier to recall. From our data we are unable to establish to which extent the identified predictors of change in diabetes management are exclusively due to the pandemic or whether they would have occurred regardless of the pandemic.

Due to the study design, certain groups have been highly represented in the study population. In particular people with type 2 diabetes and people who have passed statutory retirement age are highly represented. Thus, the study data does not offer opportunity for in depth analyses of people in specific life-situations, such as being responsible for small children and a full-time job while managing diabetes.

In conclusion, this study confirmed that many people with diabetes experienced difficulties in managing diabetes as usual during the pandemic, although the majority did not experience any difference. An important predictor for both deterioration and improvement in diabetes management was diabetes distress. Thus, diabetes management should be of concern during times of crisis and particular attention should be devoted to people with high levels of diabetes distress, who will be at increased risk of experiencing difficulties in managing diabetes.

## Data Availability Statement

The original contributions presented in the study are included in the article/**Supplementary Material**. Further inquiries can be directed to the corresponding author.

## Ethics Statement

The study was reviewed and approved by the Danish Data Protection Agency. Participants provided written informed consent prior to participating in the study.

## Author Contributions

KO, LJ, KM and IW designed the study. KM prepared data for analysis and KO carried out the statistical analyses. Interpretation of the findings was discussed among all authors. KO drafted the manuscript and all authors contributed to the final version of the manuscript.

## Acknowledgments

We wish to give thanks to the respondents for their participation in the study, and to Lone Holm and Andrea Aaen Petersen (Steno Diabetes Center Copenhagen) and Kasper Arnskov Nielsen (Danish Diabetes Association) for their indispensable efforts in participant recruitment. Finally, we would like to thank the research team at the University of Copenhagen behind ‘Standing together – at a distance’ whom the survey was developed in close collaboration with.

## Conflict of Interest

The authors declare that the research was conducted in the absence of any commercial or financial relationships that could be construed as a potential conflict of interest.

## Publisher’s Note

All claims expressed in this article are solely those of the authors and do not necessarily represent those of their affiliated organizations, or those of the publisher, the editors and the reviewers. Any product that may be evaluated in this article, or claim that may be made by its manufacturer, is not guaranteed or endorsed by the publisher.
