# Supplementary material for: How has the COVID-19 Pandemic Affected Diabetes Self-Management in People With Diabetes? - A One-Year Follow-Up Study
Source: Front Clin Diabetes Healthc. 2022 Aug 17;3:867025. doi: 10.3389/fcdhc.2022.867025 (PMC10012120; doi:10.3389/fcdhc.2022.867025)
Supplement: Supplementary file 1 [file DataSheet_1.docx]

Supplemental material: Outcome questionnaire items

Compared to before the coronavirus pandemic, how has your diabetes management changed?

|  | Significantly harder to manage (1) | Moderately harder to manage (2) | Slightly harder to manage (3) | No impact (4) | Slightly easier to manage (5) | Moderately easier to manage (6) | Significantly easier to manage (7) |
| --- | --- | --- | --- | --- | --- | --- | --- |
|  |  |  |  |  |  |  |  |

Compared to before the coronavirus pandemic, how would you describe your current diet with regard to your diabetes?

|  | Much less healthy (1) | Moderately less healthy (2) | Slightly less healthy (3) | No change (4) | Slightly healthier (5) | Moderately healthier (6) | Much healthier (7) |
| --- | --- | --- | --- | --- | --- | --- | --- |
|  |  |  |  |  |  |  |  |

Compared to before the coronavirus pandemic, are you exercising:

|  | Much less (1) | Moderately less (2) | Slightly less (3) | No change in exercise (4) | Slightly more (5) | Moderately more (6) | Much more (7) |
| --- | --- | --- | --- | --- | --- | --- | --- |
|  |  |  |  |  |  |  |  |

| Page Break |  |
| --- | --- |

Compared to before the coronavirus pandemic, how would you describe your diabetes medication taking now?

|  | I am taking my diabetes medications a lot more regularly (1) | I am taking my diabetes medications a little more regularly (2) | No change (3) | I am taking my medications a little less regularly (4) | I am taking my medications a lot less regularly (5) |
| --- | --- | --- | --- | --- | --- |
|  |  |  |  |  |  |

Compared to before the coronavirus pandemic, have you noticed a change in the following?

|  | Much less frequent | Less frequent | Slightly less frequent | No change | Slightly more frequent | More frequent | Much more  frequent |
| --- | --- | --- | --- | --- | --- | --- | --- |
|  |  |  |  |  |  |  |  |

High BG levels (1)

BG variability (2)

Low BG levels (3)
